# Identification of functionally related genes using data mining and data integration: a breast cancer case study

**DOI:** 10.1186/1471-2105-10-S12-S8

**Published:** 2009-10-15

**Authors:** Ettore Mosca, Gloria Bertoli, Eleonora Piscitelli, Laura Vilardo, Rolland A Reinbold, Ileana Zucchi, Luciano Milanesi

**Affiliations:** 1 Istituto Tecnologie Biomediche, Consiglio Nazionale Ricerche, Via Fratelli Cervi 93, 20090, Segrate (MI), Italy; 2 Max Planck Institute, D48149 Muenster, Germany

## Abstract

**Background:**

The identification of the organisation and dynamics of molecular pathways is crucial for the understanding of cell function. In order to reconstruct the molecular pathways in which a gene of interest is involved in regulating a cell, it is important to identify the set of genes to which it interacts with to determine cell function. In this context, the mining and the integration of a large amount of publicly available data, regarding the transcriptome and the proteome states of a cell, are a useful resource to complement biological research.

**Results:**

We describe an approach for the identification of genes that interact with each other to regulate cell function. The strategy relies on the analysis of gene expression profile similarity, considering large datasets of expression data. During the similarity evaluation, the methodology determines the most significant subset of samples in which the evaluated genes are highly correlated. Hence, the strategy enables the exclusion of samples that are not relevant for each gene pair analysed. This feature is important when considering a large set of samples characterised by heterogeneous experimental conditions where different pools of biological processes can be active across the samples. The putative partners of the studied gene are then further characterised, analysing the distribution of the Gene Ontology terms and integrating the protein-protein interaction (PPI) data. The strategy was applied for the analysis of the functional relationships of a gene of known function, Pyruvate Kinase, and for the prediction of functional partners of the human transcription factor TBX3. In both cases the analysis was done on a dataset composed by breast primary tumour expression data derived from the literature. Integration and analysis of PPI data confirmed the prediction of the methodology, since the genes identified to be functionally related were associated to proteins close in the PPI network. Two genes among the predicted putative partners of TBX3 (GLI3 and GATA3) were confirmed by *in vivo *binding assays (crosslinking immunoprecipitation, X-ChIP) in which the putative DNA enhancer sequence sites of GATA3 and GLI3 were found to be bound by the Tbx3 protein.

**Conclusion:**

The presented strategy is demonstrated to be an effective approach to identify genes that establish functional relationships. The methodology identifies and characterises genes with a similar expression profile, through data mining and integrating data from publicly available resources, to contribute to a better understanding of gene regulation and cell function. The prediction of the TBX3 target genes GLI3 and GATA3 was experimentally confirmed.

## Background

The identity of each cell in a multicellular organism is determined by the unique gene-expression patterns of that cell type and is specified by a complex system characterised by intricate molecular circuits. Within these networks, regulatory elements control and modulate RNA and protein expression levels. The application of the Systems Biology approach holds great promise for the identification of the structure and dynamics of cellular pathways [1], thus facilitating the understanding of the complexity associated with cellular functions. However, only a small part of these pathways has been characterised in such a way to enable them to be useful for mathematical modelling and predicting *in vivo *dynamics.

In recent years, the wide use of various high-throughput technologies has generated a large amount of data regarding the transcriptome and the proteome states of cells. Part of these data is stored in publicly available databases, such as: the Gene Expression Omnibus [2], ArrayExpress [3] and The Stanford Microarray Database (SMD) [4] that collect microarray experiments data; the Human Protein Reference Database (HPRD) [5] and the BioGRID [6] that store protein-protein interaction (PPI) data. Data collected in such resources can be integrated allowing the collection of different types of information that can be useful for strategies that aim to understand regulatory unit interactions and cellular pathways dynamics.

The comparison of gene expression profiles can be used to predict whether a number of genes are functionally related. The hypothesis is that if two genes have a similar expression profile across many biological samples then they may be functionally related. Indeed, expression profile similarity in a large number of experimental conditions is an empirical evidence that the considered genes could establish some relations to determine cell functioning. The relationship can be the involvement in the same biological process or the physical interaction between proteins. Hence, the analysis of the RNA expression levels supported by the integration of PPI data helps the reconstruction of cellular pathways.

The co-expression analysis approach was recently applied to many human microarray datasets, revealing functionally coherent groups of genes [7] and was used to create a database of co-expressed gene networks in mammals [8]. The measure of the similarity among pairs of expression profiles was based on the correlation, that permits the identification of a profile of similar shape regardless of the expression level. However, the application of co-expression analysis on datasets including samples generated with heterogeneous experimental conditions may cause some issues. In this context, it is important to adopt a strategy that considers subsets of samples. Indeed, some of the collected samples may be not relevant for the pair of genes analysed because the genes may not be active in some sample types. Moreover, two genes may be positively correlated in some conditions while negatively correlated in others.

In this work, we present a strategy based on data mining and data integration to identify genes functionally related to each other through a biological pathway or a physical interaction. The calculation of the similarity of gene expression values over a number of samples is coupled within an optimisation system that identifies the subsets of samples where the correlation between the expression profiles is the highest. The pipeline integrates data from the Gene Ontology (GO) project [9] and PPI databases in order to characterise the putative partners of the gene of interest and the type of relationship that they establish. We report the results of two applications of the presented methodology: one, for validation purposes, concerns Pyruvate Kinase (PK), while the other is related to the prediction of the putative partners of the gene T-Box 3 (TBX3), a transcription factor with the T-box DNA binding domain that is involved in the regulation of developmental processes.

## Results and discussion

### Coupling data mining and data integration for the identification of functionally related genes

**Figure 1:**
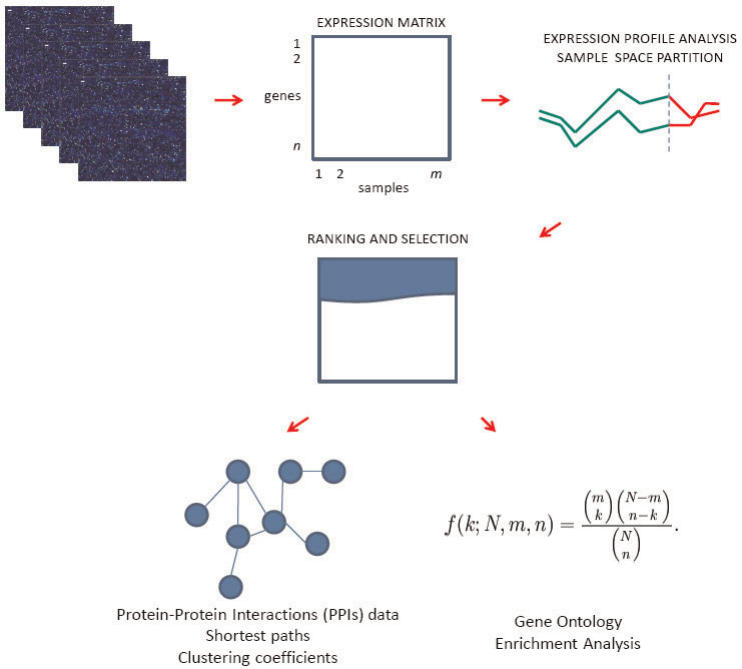
**Illustration of the pipeline**. The methodology takes as input an expression matrix. The optimisation process calculates the strength of the correlation for each gene with a chosen gene and identifies the samples where the correlation is optimal. Subsequently, genes are ranked and a number of genes are selected. Then, the selected genes are characterised analysing the GO terms enrichment and the properties of the PPI network established by the proteins they produce.

The methodology, represented in Figure 1, takes as input a genes-by-samples expression matrix *X*, where the element *x*_*i*, *j *_is a measure of the gene *i *expression in the sample type *j*.

The first part of the procedure calculates the extent of the correlation between the expression profiles of the studied gene and all the other genes in *X*. The calculation of a gene pair correlation is coupled within the optimisation process. This procedure is based on a genetic algorithm that, for each gene pair, partitions the samples into three disjoint sets such that the set *C*_+ _contains the samples in which the correlation between the genes is positive, *C*_- _contains the samples in which the correlation is negative and *C*_0 _contains the samples in which there is no correlation. The first and the second sets can be empty but at least one of them must have a size larger than a predefined threshold (under which the number of samples is considered too small to support a significant correlation analysis); the third group has a predefined limited size that represents the maximum number of samples that can be considered not relevant. Subsequently, genes are ranked according to the correlation values that they exhibit with respect to the gene of interest. The strategy implements different ranking possibilities. Indeed, it is possible (i) to focus the attention on the genes showing the highest positive or negative correlation, (ii) to produce a unified list or (iii) to consider the genes that show a dual behaviour, that is the genes that are positively correlated with the gene of interest in some samples and negatively correlated in other samples. The decision on how to do the ranking is left to the user since it is related to the specific case study.

Once the ranking is done a number of top ranked genes are selected as input for the subsequent part of the analysis. Regarding this selection, the set of genes within the first percentile constitutes a reasonable choice since its elements encode for proteins that are close to each other in the PPI network (this evidence is discussed later in the text).

The analysis follows with the integration of the GO terms and PPI data. GO terms are used to provide a list of potential components, functions and processes that are significantly enriched in the selected genes. This task is accomplished comparing the distribution of the GO terms among the selected genes with a reference distribution. In particular, the system searches for over- and under-represented terms. Due to this calculation, it is possible to obtain an initial idea of the biological role of the studied gene.

PPI data are integrated from HPRD and BioGRID. The strategy builds the network by establishing the products of the selected genes along with their first neighbourhood in the PPI network (the first neighbourhood of a node in a graph is the set of nodes that are directly connected with it). The system calculates the all-pairs shortest paths length between the proteins included in the considered network and their clustering coefficients. The shortest path between two nodes of a graph is the shortest way to walk from a node to the other following the connections between nodes; the length of the shortest path is the number of edges included in it. The clustering coefficient is a measure of the neighbourhood density of the considered node [10]. The information gained from the analysis of the shortest paths and clustering coefficients is used following the evidence that the shorter the distance between two proteins in the PPI network, the higher the probability that they have a similar function [9]. By the integration and analysis of PPI data the system determines, first of all, the subset of the functional relationships among the selected genes that are constituted by physical interactions between the encoded proteins. Moreover, it provides a list of modules of interacting proteins encoded in a format that can be used as input for software like Cytoscape [11] that allows network visualisation and analysis. These modules contain proteins that are close in the PPI network, participate in the same biological processes and in some cases could represent protein complexes.

### Breast cancer case study: breast primary tumour expression data from SMD

A dataset composed by breast cancer expression data was assembled using data available in the literature. Indeed, we were interested in gaining some insight into the potential functional partners of the Tbx3 protein in breast cancer or breast pathologies. In humans, mutations in TBX3 result in the Ulnar-Mammary Syndrome (UMS), an autosomal dominant disease that is characterised by mammary gland hypoplasia and other congenital anomalies, suggesting that TBX3 is required for normal breast development [12]. In addition, TBX3 was found over-expressed in breast cancer tissues and cell lines [13,14]. Therefore, since the level of expression of TBX3 is lower in non-cancer breast epithelial cells with respect to breast malignant cells, in order to identify genes co-regulated with TBX3 in breast cancer, we used expression data generated from breast primary tumour samples. The dataset was downloaded from SMD and includes 413 samples (Additional File 1) annotated in the database as breast primary tumours. Data were retrieved as preprocessed log_2 _fold changes, representing expression variations with respect to the reference conditions used in the experiments in which samples were collected. In order to validate both the methodology and the considered dataset, the pipeline was applied to a gene of known function, PK. The protein encoded by this gene catalyses the transphosphorylation of phosphoenolpyruvate producing pyruvate and ATP and it constitutes the last step of the glycolytic pathway.

### Genes within the top percentiles of the ranked lists are enriched in proteins that are near to each other in the PPI network

**Figure 2:**
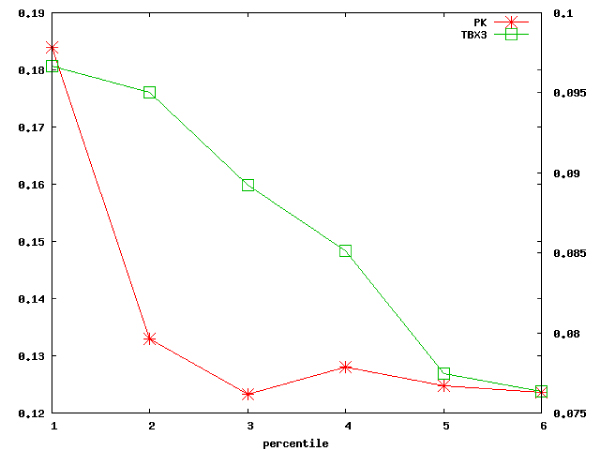
**Protein pairs closeness in the PPI network**. The plot shows the fraction of protein pairs that are close to each other in the PPI network (*d *≤ 3, see the text for details) respect to all the protein pairs within the considered percentiles of the ranked genes lists regarding the case studies of PK (asterisks, left scale) and TBX3 (squares, right scale).

In order to empirically choose a subset of the ranked gene list produced after the optimisation process, we analysed the distribution of the PPI among the proteins encoded by the ranked genes. Following the evidence that proteins that are near to each other in the PPI network have a similar function [15] we expect that if the genes within the top percentiles of the list are composed by proteins functionally related we would find that the proteins that they encode are separated by a short distance in the PPI network. Recently, it has been shown that the average distance of protein pairs in the human PPI network is 4.85 [16]. Therefore, we considered a pair of proteins (*A*, *B*) to be near if *d*(*A*, *B*) ≤ 3 where *d *is the distance between (*A*, *B*) in the PPI network. We calculated the proportion of the protein pairs with *d *≤ 3 with respect to all the possible pairs in a chosen percentile. In both the applications of the presented strategy, we found that the portion of protein pairs that are near to each other in the PPI network is the highest in the first percentile and decreases considering the genes included in the subsequent percentiles, Figure 2.

This result has a dual implication. First, it constitutes a validation for the presented methodology, and in particular for the optimisation process that evaluates the expression similarity. Indeed, its ranked output reproduces the evidence that functional similarity should correspond to closeness in the PPI network. Second, the analysis of the trend of the portion of protein pairs that are close in the PPI network with respect to the percentiles of the ranked list enables one to choose a cutoff for the selection of the genes for subsequent analyses.

In both the cases of PK and TBX3, the genes included in the first percentile were selected as the final gene list, since they exhibited the highest portion of proximal protein pairs.

### Pyruvate Kinase functionally related genes

The output of the optimisation process (Additional File 2) was ranked considering both positive and negative correlation coefficients. The analysis of the GO term enrichment among the selected genes revealed that they are involved in metabolic processes, cell cycle and response to DNA damage (Table 1). The presence of genes that participate in metabolic processes is expected since PK has a key role in metabolism. Moreover, it seems reasonable to find genes involved in cell cycle, considering that the dataset concerns breast cancer and proliferating tumour cells where expression of PK is crucial [17]. Indeed, since highly proliferating cells require energy, glycolysis is a major pathway involved in energy production.

**Table 1:** Characterisation of the genes correlated with PK

GO term id	GO term description	*p*-value
GO:0044237	cellular metabolic process	2.73E-016
GO:0044238	primary metabolic process	4.95E-015
GO:0009058	biosynthetic process	5.15E-12
GO:0007049	cell cycle	1.25E-10
GO:0034960	cellular biopolymer metabolic process	8.02E-09
GO:0034984	cellular response to DNA damage stimulus	5.28E-09
GO:0033554	cellular response to stress	5.28E-08
GO:0006260	DNA replication	7.56E-08

List of the most significant GO terms that are over-represented among the genes identified to have an expression profile correlated with that of PK.

Response to DNA damage is a process that is tightly coupled with cell cycle. Due to the unique nature of how some tumour cells grow in acute cycling and hypoxic conditions and that tumour cells have elevated rates of glucose uptake but reduced rates of oxidative phosphorylation, the unique cellular metabolism found in cancer cells may be correlated in part with the function of genes involved in DNA damage response, for instance, BRCA1 which may have a role in regulating genetic instability in hypoxic cells [18]. In order to test for a potential biological role of genes positively and negatively correlated to PK, the GO terms enrichment was evaluated separately in the two subsets (data not shown). The analysis revealed that the positively correlated genes are mainly involved in metabolism, cell cycle and DNA repair. This reflects the direct functional relationship among the processes listed above under tumour conditions. Conversely, we found that the top ranked genes negatively correlated with PK are enriched in GO terms related to "response to stimulus" and "immune system processes". It is well established that there is a relationship between cancer cells and the immune system for regulating tumour dormancy, tumour survival and tumour progression [19]. Hence, the evidence of a relationship of negative correlation between PK and genes involved in immune system processes can be the consequence of the necessity to inhibit the immune system to allow cell growth.

However, it is important to consider that the RNA used to generate the expression data in the analysed dataset was usually not derived from microdissected breast tumour cells. This implies that the RNA of different cell types can be present in the samples used for analysis and, since these cells have not been excluded, it is possible that the reported functional relationships include genes that are specific of immune system cell types.

**Figure 3:**
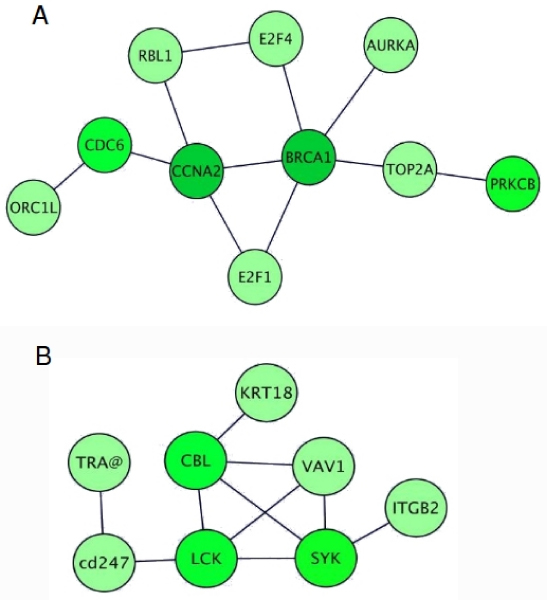
**Modules of physically interacting proteins that have a similar expression profile**. Modules of interacting proteins found within the first percentile of the ranked gene lists during the identification of genes functionally related to PK (a) and TBX3 (b). The colour gradient reflects the normalised correlation coefficient value associated with the gene: the darker the colour the higher the coefficient. Representations of the modules were generated using Cytoscape [11];

The integration and the analysis of the PPI established by the proteins encoded by the selected genes revealed a number of modules of interacting proteins. One of these (Figure 3A), includes proteins with a role in DNA replication and repair. All these proteins are encoded by genes that were reported to be positively correlated with PK.

Globally, the results described above constitute another validation of the methodology that is successfully able to identify a subset of genes that share some functional relationships with the gene of interest under analysis.

### Prediction and experimental validation of TBX3 functional partners

The output of the optimisation process (Additional File 3) was ranked considering both positive and negative correlation coefficients. The GO terms distribution was studied to search overrepresented terms among the selected genes. The terms with the most significant *p*-values are associated with processes, functions and components that are consistent with some predicted biological roles of TBX3 (Table 2). The 20 top ranked genes (Table 3) are involved in transcription regulation with a particular role in developmental processes (such as GATA3, GLI3 and PBX1), in signal transduction (such as RGL2) and in regulation of apoptosis (CFLAR and BCL2). It seems reasonable that TBX3 could regulate a network of transcription factors, generating the amplification of its signal by activation or repression of a cascade of other genes involved in the control of DNA transcription. Another class of possible TBX3 targets is the group of genes involved in cell signalling and cell communication, like membrane proteins, G-coupled membrane receptor or signal transduction proteins, as PRRT2 or RGL2, and transport proteins, as SH3BP4. In this class of genes, for instance, we reported a gene that encodes for a calcium dependent membrane protein, FSTL5, involved in the process of cellular adhesion. It is not surprising that TBX3 could control genes of the cell cycle or apoptosis, which are processes in the tumour development.

**Table 2:** Characterisation of the genes correlated with TBX3

GO term id	GO term description	*p*-value
GO:0050793	regulation of developmental process	1.65e-06
GO:0048519	negative regulation of biological process	1.86E-05
GO:0007165	signal transduction	2.21E-05
GO:0007154	cell communication	2.50E-05
GO:0002376	immune system process	3.49E-05
GO:0030528	transcription regulator activity	72E-04
GO:0051093	negative regulation of developmental process	2.43E-04
GO:0042981	regulation of apoptosis	2.97E-04
GO:0043066	negative regulation of apoptosis	4.10E-04

List of the most significant GO terms that are over-represented among the genes identified to have an expression profile correlated with that of TBX3.

**Table 3:** First 20 top ranked genes correlated to TBX3

Rank	Gene Symbol	r'	r
1	GATA3*	9.14	0.727
2	TBC1D9	8.99	0.729
3	PATZ1	8.86	0.786
4	PRRT2	8.83	0.784
5	COG2	8.79	0.723
6	RGL2	8.78	0.696
7	MAGED2	8.76	0.725
8	BCL2	8.71	0.733
9	GLI3*	8.68	0.716
10	THSD4	8.66	0.732
11	MED13L	8.64	0.706
12	PBX1	8.63	0.712
13	FSTL5	8.62	0.739
14	CGN	8.62	0.711
15	CTSS	-8.62	-0.706
16	SH3BP4	8.60	0.704
17	ARSI	8.58	0.715
18	SIN3A	8.58	0.718
19	RHBDF1	8.55	0.676
20	CFLAR	-8.55	-0.703

The table lists the first twenty top ranked genes along with their normalised *r' *and raw *r *correlation coefficients. The asterisk (*) indicates the experimental validation of the predicted relationship.

Also in the case of TBX3, the genes found negatively correlated are enriched with GO terms related to immune system processes. The interpretation of this correlation might be similar to that observed for PK in that the samples used for the analysed data set may not be derived from microdissected cells but may consist of several different cell types.

The integration and the analysis of PPI data revealed a number of modules of interacting proteins. One of these (Figure 3B) includes proteins that have a role in immune system processes.

#### Sequence based prediction and experimental validation of TBX3 transcription regulated targets

Since TBX3 is a transcription factor, it is expected that a subset of highly correlated genes identified with the presented approach, could be TBX3 gene targets or protein co-factors. Indeed, since correlation does not imply causation, TBX3 highly correlated genes are not necessarily TBX3 direct targets.

In order to find the TBX3 regulated targets, an independent computational analysis was carried out to identify the TBX3 Transcription Factor Binding Sites (TFBSs) on the genome. The analysis was done using ReXSpecies [20], a tool that analyses sequences using data from multiple species.

We found that 2 genes within the top ten ranked genes after the optimisation process have the TBX3 TFBS upstream to their gene transcription start site. They are GATA binding protein 3 (GATA3), the top ranked gene, and GLI-Kruppel family member 3 (GLI3), Table 3.

**Figure 4:**
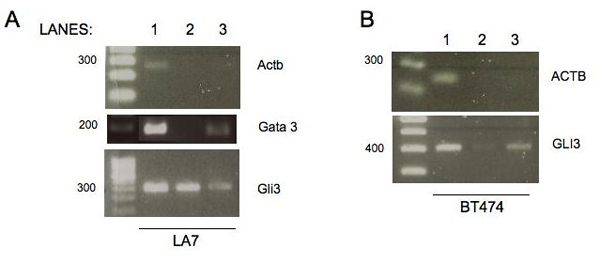
**ChIP analysis results revealed that TBX3 transcription factor regulates GATA3 and GLI2/3 target genes**. PCR amplification on LA7 cells (panel A): input samples (total genome, lane 1), preimmune sera (PI, lane 2) and chromatin immunoprecipitated material (lane 3). The expected GLi3 (rat) or GLI3(human) bands were obtained in LA7 or BT474 cells (panels A and B). Beta actin (ACTB for human; Actb for rat) was used as control in both panels.

These two genes have not been previously described to be targets of TBX3 (the only Tbx3 reported target genes are p14ARF [21] and the serum response factor, SRF [22]). The two putative TBX3 targets where validated using formaldehyde-mediated DNA-protein crosslinking [23]. DNA-protein interactions can be probed *in vivo *using an approach whereby DNA-bound proteins are first cross-linked to the DNA by treatment with formaldehyde that results in instantaneous formation of cross-linked networks, preventing transient redistribution of the cellular components. The formaldehyde-crosslinked chromatin is sonicated to introduce random and limited breakage. Then the DNA-bound protein region of the chromosome can be enriched for by immunoprecipitation with specific antibodies. The protein bound DNA regions can then be amplified and thereby validated with the use of specific primers designed to encompass the putative TBX3 protein binding site. This type of analysis can be used to identify putative TBX3 protein interaction regulatory regions and enhancer sequences on chromatin that modulate cell function [23]. This assay was performed using two different cell lines, the LA7 cell line [24-26] and a well known human breast cancer cell line, BT474. Primer pairs were selected to amplify beta-actin which was used as a negative control for both cell lines (Figure 4A and Figure 4B, upper left and right panels), and GATA3 (Figure 4A, middle panel) or GLI-Kruppel family member (GLI3) (Figure 4A and Figure 4B, lower left and right panels) regions containing the potential binding sites for TBX3 protein. As Figure 4 shows, we performed PCR amplification on total genomic DNA as a positive control of the PCR products (lane 1); in the PCR on pre-immune (PI) sera, no band was amplified as expected by the fact that chromatin should not be precipitated by the PI sera (lane 2). PCR on the chromatin DNA immuno-precipitated with TBX3 antibody confirmed that Tbx3 protein binds in vivo to Gli3 and Gata3 in LA7 cells. and GLI3 in BT474 cells (lane 3).

## Conclusion

The presented methodology identifies functionally related genes analysing a large dataset of microarray experiments and integrating data from literature. During the evaluation of a pair of expression profiles the system identifies the sample subsets where the correlation is the highest. This feature is important especially when the datasets consist of a large number of samples collected in different experimental conditions, since the pool of active biological processes depends on these conditions and hence not all the samples can be useful for the expression similarity calculation.

A dataset containing primary breast cancer expression data was assembled from the literature and used to search genes functionally related to a gene of known function, PK, and for the prediction of TBX3 functional partners. In both cases the methodology found a number of genes that share functional relationships with the gene under analysis. The integration of PPI data provided a validation of the results, confirming that genes ranked within the top percentiles encode proteins that are close in the PPI network, an evidence of functional relationship. Moreover, the analysis of PPI data enabled the identification of interacting protein modules among the putative partners of the gene of interest. In order to prove the prediction of the methodology concerning the TBX3 gene, we used a combination of chromatin immunoprecipitation (ChIP) and Polymerase Chain Reaction (PCR) analysis, a technique that is used to validate *in vivo *DNA-binding protein locations on chromatin DNA. The cell-type-specific transcription program is controlled by the availability of a set of transcription factors and the accessibility of their target sites in the chromatin. Target-site accessibility is modulated by multiple factors, including DNA methylation and histone modifications. By this technique we validated two main TBX3 target genes, GLI3 and GATA3. GATA3 is one member of a transcription factor family that plays an essential role in the specification of and maintenance of differentiated cell types, in particular in mammary gland differentiation (for review see [27,28]) and in breast cancer has emerged as a strong predictor in tumour differentiation [29,30]. It is not thus surprising that the transcription factor Tbx3 [14], being involved in tumour progression, could control the GATA3 transcription factor. It has already been reported a clear role for GLI3 transcription factors in the normal mouse mammary gland development [31] and GLI3 may have a particular role in the breast cancer metastasis to bone *in vivo *[32] as GL3 is involved in mediating epithelial-stromal tissue interaction [31].

In conclusion, the presented strategy is demonstrated to be a useful tool to mine large datasets available in the literature in order to identify functional and physical relationships established by the gene of interest and to characterise its biological role starting from the study of the available information related to its predicted partners.

## Methods

### Expression matrix preprocessing

The expression matrix preprocessing done by the methodology presented here handles the issue of the potential presence of multiple expressed sequence tags (ESTs) that represent the same gene. The pipelines supports different strategies for this task. The decision of which one to apply depends on two kinds of considerations which result in contrast outcomes: one related to molecular biology and the other related to computational time. Indeed, the possibility to include in the analysis the whole set of ESTs related to a gene has been taken into account for alternative splicing but, on the other hand, this solution is the most expensive in terms of computational cost. In the opposite case, in which only one EST is selected to represent the associated gene, the computational cost is the lowest, but the potential different behaviour of the splicing variants is lost.

Both in the cases of PK and TBX3, the EST with the higher number of available expression values across the samples was selected to represent the associated gene. For all the other genes the EST with the highest number of expression values available in the same samples with respect to the gene under analysis (PK or TBX3) was selected to represent the related gene.

### The optimisation process

Similarity between a pair of expression profiles is assessed using the Pearson's *r*. In order to enable the comparison between Pearson's coefficients calculated on a different number of pairs of expression values we use  since the statistical significance of the Pearson's *r *is proportional to the sample size *N*, that is the bigger the sample size the smaller the correlation expected by chance.

The optimisation process is handled using a Genetic Algorithm (GA). An individual is defined as a string *s *over the search space *S *∈ {+, -, 0}^*m *^that represents the possible assignments of every sample 1, 2,..., *m *to one of the sample sets *C*_+_, *C*_-_, *C*_0 _defined above. The fitness function is defined such that it has its maximum value when the samples partition determines the highest positive correlation in the set *C*_+_, the highest negative correlation in *C*_- _and no correlation in *C*_0_. Hence, the aim of the optimisation process is the maximisation of the fitness function that we defined as a map from the normalised correlation coefficients associated at each set to the interval [0, 1] ∈ ℜ:



where  is the number of samples assigned to *C*_*i*_. In particular, we used



that have, by definition, the global maximum equal to *α *= /*m*, and where . The results concerning the case studies of PK and TBX3 were obtained running the GA with a population of 500 individuals for 5000 generations.

### GO terms enrichment and PPI analyses

The systems identifies the GO terms that are under/over-represented in a given list of significant genes respect to a reference gene set. The probability that the observed distribution is obtained by chance is calculated using the appropriate cumulative hypergeometric distribution.

PPI data are integrated from HPRD and BioGRID. Given a list of proteins in input the system builds a graph that represents the PPI in which the considered proteins are involved. In particular, the nodes of the graph include the proteins given in input along with their interacting partners (their first neighbourhood); the edges are all the connections available in the databases listed above among the considered nodes. The all-pairs shortest paths are calculated using the Floyd-Warshall algorithm.

### Software

The pipeline relies on a series of Perl scripts, C programs, and MySQL databases. Considering the main tasks, data preprocessing and GO enrichment analysis rely on Perl scripts; the optimisation process and the all-pairs shortest-paths problem are solved with C programs; local MySQL databases were created to integrate data from the GO project, HPRD and BioGRID. The programs composing the pipeline are available upon request.

### Prediction of TBX3 TFBSs

TFBSs were predicted using a tool that searches genomic regions from multiple species [20]. TFBSs may be located upstream or downstream of known genes, or be part of their UTRs (untranslated regions). Typically the tools use TFBS models represented by Hidden Markov Models (HMM, used by Mapper [33]), Position Specific Weight Matrices (PWM, used by Genomatix [34]), or IUPAC consensus sequences (Genomatix) to predict TFBSs in a DNA sequence, or source of models such as Jaspar [35] and Transfac [36]; Genomatix uses a database of TFBS developed in-house. The DNA motif that was used is designed to match usually short sequences (about 8–20 base pairs). The ReXSpecies tool is designed to remove false positive matches. ReXSpecies is an evolutionary approach for TFBS prediction by phylogenetic footprinting, and is based on the idea that the sequences coding a regulatory element should be preserved across different species. The phylogenetic footprinting method tries to discover TFBS in a set of orthologous regulatory regions from multiple species, by identifying the best conserved motifs in those orthologous regions.

### TFBSs in genomic data sequence data. Experimental confirmation of TBX3 interacting partners

Validation of putative TBX3 targets was performed by formaldehyde-mediated DNA-protein cross-linking [23]. DNA-protein interactions were probed using primers designed to span the region where the putative Tbx3 binding sites were found by genomic sequence analysis. The following primers amplifying the same genes in both rat and human sequences were used:  FW 5'-TTCTCACTGGTTCTCTCTTCTGCC-3' and RW 5'-TTGGGATGGGGAGTCTGTTCAG-3' amplifying a fragment of 310 bp from the beta Actin gene of rat (Gene ID N. 81822) and human (Gene ID N. 60); amplifying a fragment of 300 bp from Gli3 gene of rat (Gene ID  140588) FW 5’-TTTTGGGAGTAGGGGCAACTG-3’ and RW 5’-TCTCCGTTCACACCTTTTGCTATC-3’ amplifying a fragment of 289 bp, and human (Gene ID 2737) FW 5’-CAGTTAGAGAGAAGGGACGGACAG-3’ and RW 5’-CAGGGTGTAAAAAAGGCATCC-3’ and amplifying a fragment of 431 bp; FW 5'-CTGGGTGAGCCACCATCA-3' and RW 5'-AGAGATCCGTGCAGCAGAG-3' amplifying a fragment of 255 bp from GATA 3 gene of rat (Gene ID 85471).

## Competing interests

The authors declare that they have no competing interests.

## Authors' contributions

EM performed the bioinformatic analysis, analysed the data, wrote the paper; GB performed research, analysed the data; EP performed research, analysed the data; LV contributed new reagents tools; RR conceived and designed the research project, analysed the data, wrote the paper; IZ conceived and designed the research project analysed the data, wrote the paper; LM contributed new analysis tools.

## Supplementary Material

Additional file 1 **List of the samples**. List of the samples retrieved from SMD and included in the analysed dataset. Click here for file

Additional file 2 **Ranked gene list, PK**. Ranked list of genes functionally related with PK. Click here for file

Additional file 3 **Ranked gene list, TBX3**. Ranked list of genes functionally related with TBX3. Click here for file
